# Métastase cérébrale d'un cancer de l'endomètre: à propos d'un cas et une revue de la literature

**DOI:** 10.11604/pamj.2015.20.68.6097

**Published:** 2015-01-26

**Authors:** Fadila Kouhen, Mohammed Afif, Mustapha El kabous, Fadoua Raiss, Naoual Benhmidou, Sanaa Majjaoui, Hanan Elkacemi, Tayeb Kebdani, Noureddine Benjaafar

**Affiliations:** 1 Service de Radiothérapie, Institut National d'Oncologie, Université Mohammed V, Rabat, Maroc; 2 Service d'Oncologie Médicale, Institut National d'Oncologie, Université Mohammed V, Rabat, Maroc

**Keywords:** Métastase cérébrale, cancer de l´endomètre, radiothérapie, Brain metastasis, endometrial cancer, radiotherapy

## Abstract

Les métastases cérébrales du cancer de l'endomètre sont rares, peu de cas ont été rapportés dans la littérature. Nous rapportons le cas d'une patiente de 62 ans qui a été traitée en 2009 pour un adénocarcinome de l'endomètre classé initialement stade Ia de la FIGO, grade 3 de l'OMS et qui a présenté deux ans après, une métastase cérébrale unique sans autres métastases à distance, traitée par une irradiation sur l'encéphale total à une dose de 30gy (10x3Gy) suivie d'une chimiothérapie à base de paclitaxel et carboplatine, avec une bonne évolution clinique et radiologique.

## Introduction

Le cancer de l'endomètre est fréquent, représente le premier cancer gynécologique en Europe et en Amérique du Nord [1] et le 4ème selon les registres de Rabat de 2007. Dans plus de 75% des cas, il est diagnostiqué à un stade localisé, expliquant ainsi son pronostic favorable avec un taux de survie à cinq ans avoisinant 80%. Les lésions métastatiques sont rares (moins de 5%) et se localisent principalement au niveau pulmonaire et hépatique. Les métastases cérébrales du cancer de l'endomètre sont très rares, et ne se voient que dans 0,3 à 1,4% des cas [2, 3], il est considéré comme un cancer neurophobe [4]. Le but de ce travail est de rapporter le cas d'une métastase cérébrale d'un cancer de l'endomètre traité à l'institut national d'oncologie à rabat avec une revue de la littérature.

## Patient et observation

**Figure 1:**
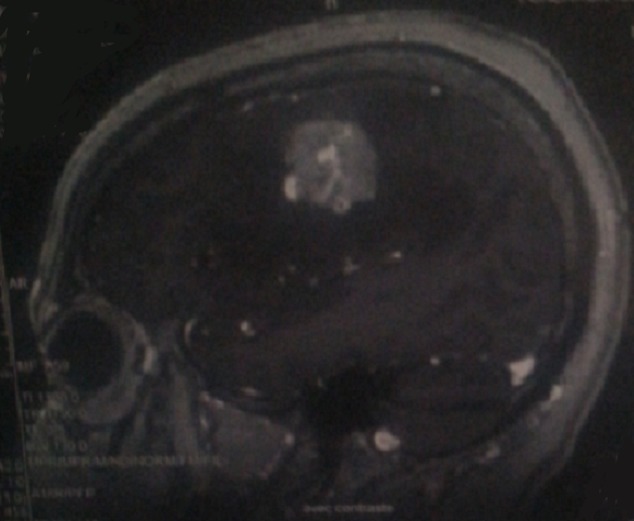
IRM cérébrale: coupe sagittale en en T2 objectivant la métastase cérébrale

Il s'agit d'une patiente âgée de 62 ans, ménopausée depuis 15 ans, traitée en 2009 pour un adénocarcinome de l'endomètre révélé par des métrorragies de moyenne abondance et une lésion éxophytique de l'endomètre à l'hysteroscopie. La patiente avait bénéficié d'une colpohystérectomie totale avec annexectomie et curage ganglionnaire bilatéral. L'examen anatomopathologique de la pièce opératoire avait objectivé un adénocarcinome endometrioide moyennement différencié et infiltrant moins de 50% de l’épaisseur du myometre, grade 3 de l'OMS. Le curage ganglionnaire était négatif (14N- / 14 N). La patiente était classée stade IA/ selon la classification de la FIGO 2009. Une radiothérapie externe adjuvante sur le lit tumoral à une dose de 46 Gy (2 Gy /Fraction) suivie d'une curiethérapie endovaginale à la dose de 2*7Gy ont été indiquées. Deux ans après la fin du traitement, la patiente a présenté des céphalées importantes rebelles au traitement symptomatique. Une tomodensitométrie cérébrale avait objectivé une lésion fronto-pariétale gauche d'allure secondaire. Un complément d'imagerie par IRM cérébrale a confirmé la présence d'un processus tumoral pariéto-occipital droit en isosignal en T1 et en hypersignal en T2 avec engagement sous falcoriel débutant évoquant une localisation secondaire (Figure 1). La patiente a bénéficié d'une biopsie stéréotaxique dont l´étude anatomopathologique morphologique et immunohistochimique était en faveur d'une métastase cérébrale d'un adénocarcinome peu différencié avec des récepteurs hormonaux positifs. Une tomodensitométrie thoraco-abdomino-pelvienne a été réalisée dans le cadre du bilan d'extension locorégional et à distance et s'est révélée normale. La patiente a bénéficié d'une radiothérapie externe sur l'encéphale total à une dose de 30Gy (3 Gy /Fraction) délivrée par deux faisceaux latéraux droit et gauche aux rayons X de 6 Mv; suivie de 6 cures de chimiothérapie à base de docetaxel et carboplatine avec une bonne tolérance clinique et biologique. La patiente est à 30 mois de la fin du traitement de sa métastase avec un bon contrôle locorégional et à distance clinique et radiologique.

## Discussion

Le cancer de l'endomètre est le cancer gynécologique le plus fréquent en occident, et le troisième cancer féminin après le cancer du sein et du colorectum [1]. Il est considéré comme un cancer hormondépendant, avec une nette prédominance chez les femmes ménopausées. Les principaux facteurs de risque sont: l’âge, la surexposition aux ‘strogènes sans exposition adéquate à la progestérone soit d'origine endogène ou d'origine exogène, le diabète. Le cancer de l'endomètre est souvent diagnostiqué à un stade limité à l'utérus, ce qui explique son excellent pronostic. La prévalence des métastases cérébrales chez les femmes atteintes de cancer de l´endomètre est de 0,3-1, 4% dans la littérature [2, 3]. Le principal mécanisme de dissémination est la diffusion des cellules cancéreuses vers les poumons, puis vers le cerveau par l´intermédiaire de l'artère pulmonaire [4–6]. A nos jours, 119 patientes sont décrites dans la littérature. L’âge des patientes ayant un cancer de l'endomètre avec des métastases cérébrales varie dans la littérature entre 48 à 82 ans avec une médiane de 66 ans [7]. Le délai de diagnostic entre la tumeur primitive et l'apparition de la métastase cérébrale est entre 0 à 52 mois [8–10]. Le délai était de 24 mois dans notre cas. Lors du diagnostic initial du cancer de l'endomètre, 36,7% ont un stade I et II selon la classification FIGO 2009, et 63,3% ont un stade III et IV, 5,5% ont un grade 1, 16,4% ont un grade 2, et 78,1% ont un grade 3. Le type histologique de carcinome de l´endomètre le plus fréquent est l'adénocarcinome endométrioide (72,4%) et 27,6% avaient d'autres types histologiques considérés comme défavorables (un carcinome adénosquameux, un carcinome à cellules claires, un carcinome séreux, un carcinosarcome). Notre patiente avait un adénocarcinome de l'endomètre stade Ia selon la classification FIGO 2009, grade 3.

Dans la littérature, 49% des patientes ont des métastases cérébrales isolées, c’était le cas de notre patiente contre 51% qui ont des métastases cérébrales dans le cadre d´une maladie disséminée qui touche également des sites extra crâniens préférentiellement le pelvis, le péritoine, le poumon, les os, le foie, et les ganglions lymphatiques [7–9]. Peu de facteurs pronostiques ont été impliqués comme étant des facteurs de risque de dissémination de la maladie et l'apparition de métastases, y compris les métastases cérébrales, il s'agit essentiellement du type histologique autre que l'adénocarcinome, le haut grade histologique, le stade avancé, et la présence d'emboles vasculaires [9–10–11]. Notre patiente avait deux facteurs de mauvais pronostic: la présence d'emboles vasculaires et le grade 3. Les recommandations actuelles pour le traitement d´une métastase cérébrale unique chez un patient avec un bon état général est la résection chirurgicale ou radiochirurgie suivie d´une irradiation de l'encéphale in toto [7–10–12]. Un traitement multimodal des métastases cérébrales (chirurgie + radiothérapie + /- chimiothérapie) montre un bénéfice en survie globale (médiane de survie de 9,2 mois) par rapport à la radiothérapie seule (survie médiane de 0,9 mois) (p = 0.0001) ou à l'abstention thérapeutique (survie médiane de 0.2 mois) (p = 0.009) [8–13]. The Radiation Therapy Oncology Group a démontré dans une étude prospective de plus de 300 patients atteints de divers cancers et une métastase cérébrale unique que le boost sur la métastase par une radiothérapie stéréotaxique après une radiothérapie sur encéphale in toto améliore significativement la survie [14]. Dans notre cas, une radiochirurgie a été proposé mais non réalisée. Pour les patientes qui présentent des métastases cérébrales multiples symptomatiques, l'irradiation de l'encéphale in toto est le traitement standard [8, 9]. La chimiothérapie à base de carboplatine et paclitaxel peut être indiquée surtout lorsque les métastases cérébrales sont dans le cadre d´une maladie disséminée. L'hormonothérapie dans le cadre de la prise en charge des cancers de l'endomètre métastatiques a des indications bien définies: adénocarcinome bien différenciée, récepteurs hormonaux positifs ou mauvais état général. La survie médiane dans la littérature est de 5mois (1-82) mois [15, 16]. La survie chez notre patiente est de 36 mois.

## Conclusion

Bien que les métastases cérébrales de cancer de l´endomètre soient rares, elles doivent être évoquées chez toute patiente avec un cancer de l'endomètre qui présente des signes neurologiques, même si la maladie est contrôlée localement et il n'y a pas d'autres sites métastatiques. Le traitement est basé essentiellement sur la résection chirurgicale ou radio chirurgie suivie d´une irradiation de l'encéphale in toto. Le pronostic est souvent mauvais surtout si la maladie est disséminée aux plusieurs sites.
